# The SCeiP Model for Remote Rehabilitation in Homebound Patients With Coronary Heart Disease

**DOI:** 10.2196/69927

**Published:** 2025-03-28

**Authors:** Siqi Zhang, Tielong Chen

**Affiliations:** 1 Hangzhou TCM Hospital of Zhejiang Chinese Medical University (Hangzhou Hospital of Traditional Chinese Medicine) Hangzhou China; 2 Department of Cardiology Hangzhou TCM Hospital of Zhejiang Chinese Medical University (Hangzhou Hospital of Traditional Chinese Medicine) Hangzhou China

**Keywords:** remote exercise rehabilitation, SCeiP model, coronary heart disease, promotion strategy, home rehabilitation

We read with great interest the study by Xu et al [1] examining the use of the “SCeiP” model to guide exercise rehabilitation in patients with coronary artery disease. The authors assessed the feasibility of a personalized exercise rehabilitation program informed by the “SCeiP” model and suggested that telehealth interventions could enhance both patients’ adherence to exercise prescriptions and their overall understanding of rehabilitation. As social workers, we attach paramount importance to the role of cardiac rehabilitation training in improving patients’ quality of life. Even with low participation rates, such interventions continue to yield significant benefits. Nonetheless, certain limitations merit further consideration.

First, the intervention group’s personalized exercise program, supported by the “SCeiP” model and facilitated through WeChat groups, exercise logs, and activity-tracking bracelets, may have been influenced by individual variations among participants. Such differences could introduce bias. Moreover, the reliance on self-reported data, often in the form of questionnaires and scales, can introduce subjectivity. Overly optimistic or pessimistic self-assessments may compromise data accuracy and obscure the true impact of the intervention.

Second, the study’s exercise intervention focused predominantly on a single form of exercise. Future research should consider incorporating a wider array of exercise modalities and exploring their synergistic effects. For instance, aerobic training [2], resistance exercises [3], and aquatic activities [4] have all shown promise in inhibiting the progression of coronary artery disease. Different exercise types may yield distinct benefits for specific patient groups. Franklin et al [5] noted that the cardiac demands of any physical activity depend on the individual’s metabolic capacity. Accordingly, future investigations could refine their methods to account for differing responses to various exercise modalities.

Third, while the study demonstrated that 1- and 3-month interventions significantly improved patients’ cognitive levels regarding cardiac rehabilitation, their sustainability and long-term clinical implications remain uncertain. Further data collection and longer follow-up periods are essential to determine whether cognitive gains translate into substantive health improvements. Additionally, the absence of subgroup analyses or baseline adjustments for variables such as age, gender, and disease severity may limit the generalizability of the results, despite the study’s use of randomized group assignments.

In conclusion, we greatly value the work of Xu et al [1], as their innovative application of the “SCeiP” model presents a new frontier for designing exercise rehabilitation programs for patients with coronary artery disease. Although further exploration is needed to diversify exercise strategies and evaluate long-term outcomes, these findings support the potential clinical and economic benefits of tele-rehabilitation technologies and offer a vital theoretical foundation for integrating the “SCeiP” model into future cardiac rehabilitation protocols.

## References

[1] Xu Dandan, Xu Dongmei, Wei Lan, Bao Zhipeng, Liao Shengen, Zhang Xinyue (2024). The effectiveness of remote exercise rehabilitation based on the "SCeiP" model in homebound patients with coronary heart disease: randomized controlled trial. J Med Internet Res.

[2] Parry-Williams Gemma, Sharma Sanjay (2020). The effects of endurance exercise on the heart: panacea or poison?. Nat Rev Cardiol.

[3] Wu Chunchun, Bu Rongsheng, Wang Yaoguo, Xu Chaoxiang, Chen Youfang, Che Lishuang, Wang Shengnan (2022). Rehabilitation effects of circuit resistance training in coronary heart disease patients: a systematic review and meta-analysis. Clin Cardiol.

[4] Faíl Luís B, Marinho DA, Marques EA, Costa MJ, Santos CC, Marques MC, Izquierdo M, Neiva HP (2022). Benefits of aquatic exercise in adults with and without chronic disease-a systematic review with meta-analysis. Scand J Med Sci Sports.

[5] Franklin Ba, Thompson Pd, Al-Zaiti Ss, Albert Cm, Hivert M, Levine Bd, Lobelo F, Madan K, Sharrief Az, Eijsvogels Tm (2020). Exercise-related acute cardiovascular events and potential deleterious adaptations following long-term exercise training: placing the risks into perspective–an update: a scientific statement From the American Heart Association. Circulation.

